# A Link-Level Simulator of the cdma2000 Reverse-Link Physical Layer

**DOI:** 10.6028/jres.108.028

**Published:** 2003-08-01

**Authors:** H. Gharavi, F. Chin, K. Ban, R. Wyatt-Millington

**Affiliations:** National Institute of Standards and Technology, Gaithersburg, MD 20899-8920

**Keywords:** cdma2000, CDMA (code division Multiple Access), IMT 2000, rake receiver, reverse link, third generation mobile systems (3G)

## Abstract

The cdma2000 system is an evolutionary enhancement of the IS-95 standards which support 3G services defined by the International Telecommunications Union (ITU). cdma2000 comes in two phases: 1XRTT and 3XRTT (1X and 3X indicates the number of 1.25 MHz wide radio carrier channels used and RTT stands for Radio Transmission Technology). The cdma2000 1XRTT, which operates within a 1.25 MHz bandwidth, can be utilized in existing IS-95 CDMA channels as it uses the same bandwidth, while 3XRTT requires the commitment of 5 MHz bandwidth to support higher data rates. This paper describes a software model implementation of the cdma2000 reverse link and its application for evaluating the effect of rake receiver design parameters on the system performance under various multipath fading conditions. The cdma2000 models were developed at the National Institute of Standards and Technology (NIST), using SPW (Signal Processing Worksystem) commercial software tools. The model has been developed in a generic manner that includes all the reverse link six radio configurations and their corresponding data rates, according to cdma2000 specifications. After briefly reviewing the traffic channel characteristics of the cdma2000 reverse link (subscriber to base station), the paper discusses the rake receiver implementation including an ideal rake receiver. It then evaluates the performance of each receiver for a Spreading Rate 3 (3XRTT) operation, which is considered as a true “3G” cdma2000 technology. These evaluations are based on the vehicular IMT-2000 (International Mobile Telecommunication 2000) channel model using the link budget defined in cdma2000 specifications for the reverse link.

## 1. Introduction

Third generation (3G) wireless systems are capable of providing high bit rate data services and can operate reliably in different types of environments: macro, micro, and pico cellular; urban, suburban, and rural; indoor and outdoor. In other words, 3G systems are expected to offer better quality and coverage, be more power and bandwidth efficient, and be deployed in diverse environments. The 3G standard consists mainly of two CDMA (Code Division Multiple Access) based systems known as cdma2000 [1] and W-CDMA (Wideband CDMA) [2]. CDMA is a multiple access method used earlier in IS-95^1^, in which a communication channel is defined as a chip^2^ sequence. In a CDMA system, each data symbol consists of a number of chips, which allows multiple users to share the same frequency band simultaneously. Communication between a mobile station (e.g., mobile phone) and a base station is two-way: the forward link and reverse link. The forward link is the transmission path from a base station to a mobile station. The reverse link is the transmission path from a mobile station to the base station.

The reverse and forward link simulation models were recently developed at the National Institute of Standards and Technology (NIST) in collaboration with Cadence Design^3^ using a simulation tool called the SPW (Signal Processing Worksystem). SPW is a system-level design tool that can allow evaluation of complex communication systems for scenarios including different channel characteristics such as path-loss, delay spread, Doppler fading, shadowing etc. The design of these models, together with a detailed report on the implementation aspects of the reverse link, can be obtained from the NIST web-site.^4^

**Fig. 1:**
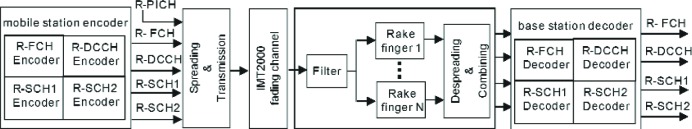
Block Diagram of the cdma2000 reverse link physical layer for traffic signal.

In this paper we are mainly concerned with the effect of the receiver design on the performance of the cdma2000 reverse link. In the following sections, we first present a brief overview of the essential components of the reverse link, according to the specifications defined by the cdma2000 standard [1], [3]. These include the functionality of the blocks shown in Fig. 1, which correspond the end-to-end transmission system. Section 2 presents the reverse traffic channels and their corresponding frame structure. Multiplexing and the transmission aspects of the cdma2000 reverse link are presented in Sec. 3. Section 4 discusses the propagation channel model. The receiver design aspects, using non-ideal and ideal rake receivers as well as diversity reception, are presented in Sec. 5. This section also includes a brief description of the channel despreader and decoder at the receiver (i.e., base station decoder). The reverse link receiver performance is measured based on its link budget specifications [1]. The link budget, which is presented in Sec. 6, emphasizes the calculation of output power for the reverse channels using open loop power estimation. The paper finally presents the results in terms of Frame Error Rate (FER) and Bit Error Rate (BER), under various test conditions in Sec. 7.

## 2. Reverse CDMA Channel Signals

The reverse link is the transmission link from a mobile station to the base station. With a CDMA technology, a mobile station can multiplex and transmit different data streams over the reverse link using several transmission channels at the same time. The channels transmitted on the reverse link consist of: Access Channel, Enhanced Access Channel, Reverse Common Control Channel, Reverse Pilot Channel (R-PICH), Reverse Dedicated Control Channel (R-DCCH), Reverse Fundamental Channel (R-FCH), Reverse Supplemental Channel 1 (R-SCH1), Reverse Supplemental Channel 2 (R-SCH2), and Reverse Supplemental Code Channels (R-SCCH). The first three channels are common channels used for MAC (Medium Access Control)^5^ messages from a mobile station to a base station [4]. The remaining five channels, i.e., the R-PICH, R-DCCH, R-FCH, R-SCH, and R-SCCH, are allocated on a per user basis and are referred to as Reverse Traffic Channels.

The focus in this paper is on the Reverse Traffic Channel, whose functions are described in Secs. 2.1–2.3. Signals transmitted on the Reverse Traffic Channel are specified by six different Radio Configurations [3]. For the reverse traffic operation in Radio Configurations 1 and 2, a single R-FCH and up to seven R-SCCHs are utilized. These two Radio Configurations are designed for backward compatibility with the existing IS-95 CDMA system. As our main concern is the cdma2000 system, Radio Configurations 1 and 2 will not be discussed in this paper.

### 2.1 Reverse cdma2000 Traffic Channels

For the reverse traffic operation in Radio Configurations 3 through 6, an R-PICH is required together with up to one R-DCCH, up to one R-FCH, and up to two R-SCHs. The following describe the channel structures, modulation parameters and other characteristics of these four Reverse Traffic Channels. In addition to Radio Configurations, the cdma2000 standard also defines the two types of spreading rates. Spreading rates 1 and 3, respectively, correspond to chip rates of 1.2288 Mcps (mega chips per second) and 3.6864 Mcps.

**Fig. 2:**
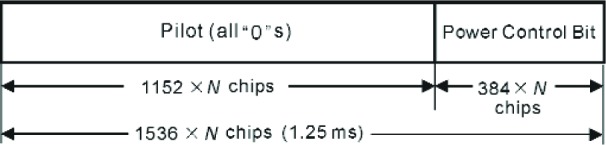
Revise Pilot Channel (R-PICH) structure.

The Reverse Pilot Channel is an unmodulated spread spectrum signal used for initial acquisition, time tracking, channel estimation, and power control. Power control is a technique to manage the transmission power to minimize interference at the base station. The R-PICH is transmitted with Radio Configurations 3 through 6. The mobile station inserts a Reverse Power Control Subchannel on the R-PICH. This subchannel carries the power control bit, which is sent to the base station for power control purposes. Each 20 ms frame in the R-PICH is divided into 16 power control groups of 1.25 ms duration. Each power control group contains 1536×*N* chips, in which pilot signal is transmitted in the first 1152×*N* chip and power control signal is transmitted in the following 384×*N* chip for spreading rate *N* (1 or 3) as shown in Fig. 2.

User voice and data traffic are transported across the reverse link on the R-FCH and R-SCHs. A single R-FCH per mobile station is permitted, providing variable data rates up to 9.6 kbit/s in Radio Configurations 3 and 5, or 14.4 kbit/s in Radio Configurations 4 and 6. The use of the R-SCHs offers two additional higher rate channels with fixed rate transmissions.

Data is processed in 5 ms or 20 ms frames. The 5 ms frame option will not be discussed in this paper. The data rate on the R-FCH can be fixed or variable during transmission. The variable rates are changed on a frame-by-frame basis. For the R-SCHs, the data rate remains constant during transmission.

The R-DCCH is used for the transmission of higher level data and control information from a mobile station to a base station. This channel can be enabled or disabled on a frame-by-frame basis. The data rate for the R- DCCH is fixed at 9600 bit/s or 14400 bit/s for 20 ms frames.

Table 1 lists the spreading rates, ratesets, and possible data rates for different Radio Configurations. The two spreading rates 1 and 3 correspond to chip rates of 1.2288 Mcps and 3.6864 Mcps respectively. Rateset defines a particular set of data rates. The data rates in Radio Configurations 3 and 5 are included in rateset 1, while the data rates in Radio Configurations 4 and 6 are in included in rateset 2. The lowest four data rates, i.e., (1500, 2700, 4800, and 9600) bit/s for rateset 1, in each Radio Configuration are permitted for the R-FCH, while all of the rates are available for the R-SCHs. The spreading rate and data rate for different Radio Configurations for the R-DCCH are also listed in Table 1.

**Fig. 3:**
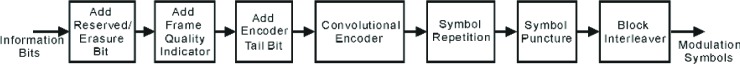
Framing and Encoding for the R-FCH, R-SCH and R-DCCH.

**Fig. 4:**
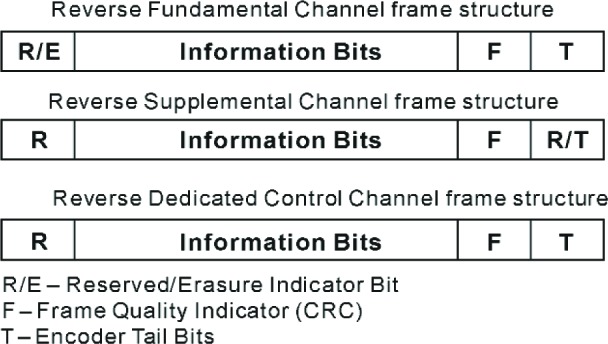
Frame Structure for the R-FCH, R-SCH, and R-DCCH.

The R-FCH, R-SCH, and R-DCCH have a common structure to generate and encode a frame. Figure 3 shows the framing and encoding procedures for these channels. The first three blocks append bits to a frame of information bits. A single reserved or erasure indicator bit may be placed prior to the information bits. The information bits with a reserved or erasure bit, are followed by the frame quality indicator and encoder tail bits. Figure 4 illustrates the order of the bits in a 20 ms frame for the R-FCH, R-SCH and R-DCCH.

The reserved/erasure indicator bit is used to indicate a bad frame on the forward link or to re-enable its transmitter after tuning to another frequency. The frame quality indicator is the CRC (Cyclic Redundancy Code) bits and is used for error detection at the receiver. The frame quality indicator covers all bits within a frame except the frame quality indicator itself and the encoder tail bits. Encoder tail bits are used to terminate the convolutional code. This ensures that the encoder returns to the initial state at the end of the frame. Once the frame is formed, it is encoded using the convolutional encoder for error protection. Convolutional code [5] is a forward error correction (FEC) code, which allows a receiver to recover the corrupted received data by exploiting the redundancy in the encoded data stream. The convolutional code rates used in the cdma2000 system are 1/2, 1/3, and 1/4 with constraint length 9 (Please refer to the Appendix A for further details).

Code symbol outputs from the convolutional encoder may be repeated based on the repetition factor. The repetition factor represents the number of times that each symbol appears right after symbol repetition. For instance, a repetition factor 2 means a symbol is repeated once. Symbol repetition plays a role in adjusting the transmission rate.

Following symbol repetition, some symbols are punctured (or deleted) as required to provide a correct rate for the modulation process. The puncturing patterns used with convolutional codes are defined in [1]. After puncturing, the symbol ordering is rearranged in the block interleaver. This is to protect groups of data from being corrupted at the same time by any deep fade or noise burst. Details of interleaver parameters for different sizes have been specified in [1].

Following the block interleaver, symbols may be repeated for further rate adjustment. In the case of R-FCH and R-SCCH with a spreading rate of 3, the repetition factor is 3. On the R-SCH, the repetition factor depends on the Radio Configuration, Walsh function (see Sec. 3.1), and data rate. As an example, Table 2 lists the parameters associated with each block in Fig. 3 on the R-FCH and R-SCH for Radio Configuration 5. Please refer to [1] for further details on the parameters in each block.

## 3. Multiplexing and Transmission

**Fig. 5:**
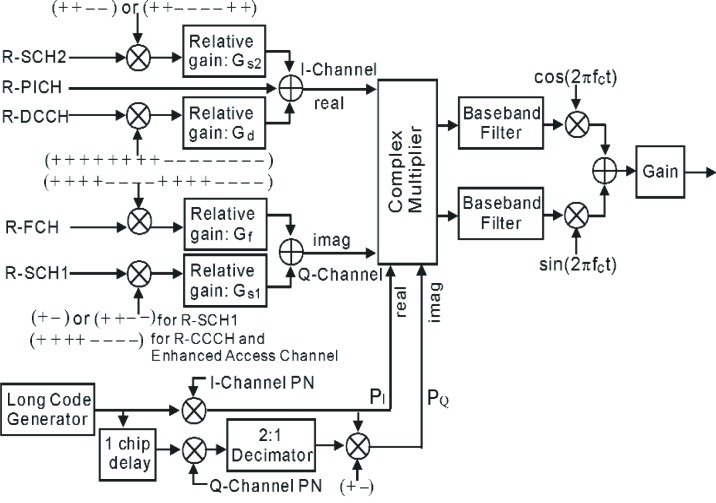
Reverse channel spreading and quadrature spreading for Radio Configuration 5.

This section describes first the manner in which the reverse channel signals are spread and combined, and then the arrangement of quadrature spreading. Figure 5 illustrates the modulation process for Radio Configurations 3 through 6. The long code generator, which will be discussed in Sec. 3.2, operates at 1.2288 Mcps for Radio Configurations 3 and 4, and 3.6864 Mcps for Radio Configuration 5 and 6. For Radio Configurations 1 and 2, the procedure is similar to that of IS-95 and is not described here.

### 3.1 Orthogonal Spreading

Orthogonal codes comprise a set of chip sequences in which each sequence is orthogonal to the others. Orthogonal codes, also known as canalization codes, are used to ensure orthogonality (e.g., independency) between the channels. Each channel is spread by an orthogonal code defined by a Walsh function [6]. The orthogonal characteristics of Walsh functions ensure that the channels do not interfere with each other. The Walsh functions are row vectors of Walsh-Hadamard matrices. Let ***W****^N^* be referred to as a *N*× *N* Walsh-Hadamard matrix, then ***W***^2^*^N^* is recursively generated from a *N*×*N* Walsh-Hadamard matrix as follows,

$$W^{2N}=\left[\begin{array}{c} W^{N}\;W^{N}\\ W^{N}-W^{N} \end{array}\right],$$

where ***W***^1^ is [ + ] and *N* is a power of 2. For instance, ***W***^4^ (4×4 Walsh-Hadamard matrix) is given by

$$W^{4}=\left[\begin{array}{cccc} + & + & + & +\\ + & - & + & -\\ + & + & - & -\\ + & - & - & + \end{array}\right],$$

where “−” is −1 and “+” is 1.

A Walsh function $W_{n}^{N}$ represents a binary (+1,−1) sequence that is a repetition of the (*n*+1)th row of an *N*×*N* Walsh-Hadamard matrix. The specific Walsh functions used for different reverse channels are listed in Table 3 As shown in Fig. 5, the Walsh functions are multiplied to the corresponding reverse channels. Further details of Walsh code assignments for traffic channels along with allowable data rates and corresponding post-interleaver repetition rates can be found in [1].

The reverse channels spread by the orthogonal codes are combined to make up a complex sequence. The real and imaginary parts of the complex sequence are referred to as the I-Channel and Q-Channel, respectively. The I-Channel consists of the R-PICH, R-DCCH, and R-SCH2, while the Q-Channel consists of the R-FCH and R-SCH1. Each reverse channel is scaled by a relative gain before the combination. The relative gains will be discussed in the link budget calculations in Sec. 6. The complex sequence is then multiplied by the quadrature spreading sequence that will be discussed in the next section.

### 3.2 Quadrature Spreading

Although reverse traffic channels are intended not to interfere with each other by orthogonal codes, in practical situations they can not maintain their orthogonalities due to the effect of filtering as well as multipath fading. Multipath fading is a propagation phenomenon of the channel variations characterized by the arrival of multiple versions of same signal due to reflection, diffraction and scattering of radio waves. The quadrature spreading sequence is arranged in a way that reduces the effect of the multipath fading and restores some of the orthogonality losses between users. The arrangement of the quadrature spreading is shown in Fig. 5. As shown in this figure, a long code, which has a period of 2^42^−1 chips, is generated by the following generator polynomial,

$$\begin{array}{l} P(x)=x^{42}+x^{35}+x^{33}+x^{31}+x^{27}+x^{26}+x^{25}+x^{22}+x^{21}\\ +x^{19}+x^{18}+x^{17}+x^{16}+x^{10}+x^{7}+x^{6}+x^{5}+x^{3}+x^{2}+x+1. \end{array}$$

The long code generator is required to generate spreading sequences at a chip rate of 1.2288 Mcps for spreading rate 1 and 3.6864 Mcps for spreading rate 3. Note that the cdma2000 system periodically sends the long code state information to user through “Sync Channel”. As shown in Fig. 5, the Quadrature components are complex multiplied by the two PN sequences, which are themselves multiplied by a long code. This is to ensure that cross correlations between the signals from distinct stations are always small.

The I-Channel and Q-Channel PN sequences are periodic with a period of 2^15^ chips for spreading rate 1 and 3×2^15^ chips for spreading rate 3. For spreading rate 1, the PN sequences are based on the following generator polynomials:

$$\begin{array}{l} P_{I}(x)=x^{15}+x^{13}+x^{9}+x^{8}+x^{7+}x^{5}+1\;(\text{I-Channel})\\ P_{Q}(x)=x^{15}+x^{12}+x^{11}+x^{10}+x^{6}+x^{5}+x^{4}+x^{3}+1\;(\text{Q-Channel}). \end{array}$$

The maximum length PN sequence that can be obtained from these polynomials is 2^15^−1. In order to obtain the I-Channel and Q-Channel PN sequence (of period 2^15^), a “0” is inserted in each sequence after 14 consecutive “0” outputs (this occurs only once in each period). Therefore, the PN sequences have one run of 15 consecutive “0” outputs instead of 14. For spreading rate 3, the PN sequences are truncated sequences of a maximal length linear feedback shift register sequence^6^ based upon the generator polynomial of *x*^20^+*x*^9^+*x*^5^+*x*^3^+1.

The I-Channel and Q-Channel PN sequences are formed from this maximal length sequence of length 2^20^−1 using different starting positions and truncating the sequences after 3×2^15^ chips. The starting position of the I-Channel PN sequence is such that the first chip is the “1” after the 19 consecutive “0”s. The starting position of the Q-Channel sequence is the starting position of the I-Channel PN sequence delayed by 2^19^ chips.

Following quadrature spreading, the resulting complex impulse sequence is passed through a transmission filter to avoid interference with adjacent frequency bands [1].

## 4. Channel Model

**Fig. 6:**
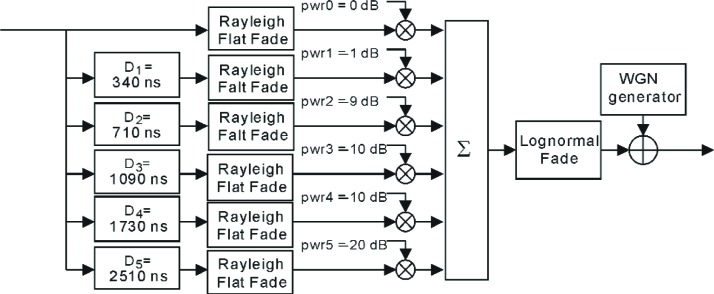
Frequency selective fading channel for the Vehicular Model-A.

The propagation channel model used in reverse link systems is that specified by IMT-2000^7^ for Vehicular model-A^8^. This model takes into account both the slow and fast fading. The slow fading is long-term variations in the received signal level and its envelope is modeled by a lognormal distribution [7]. The slow fading is also referred to as shadowing. The fast fading is short-term variations in the received signal level and is modeled by the superposition of multiple paths with different average powers and arrival times. The average power and arrival time are assumed to be fixed and are determined by the channel impulse response. Each path has a Rayleigh distribution, with the power spectrum suggested by Jakes [7]. Figure 6 shows a six-path frequency selective fading channel that has been used for the reverse link. Further details on the channel modeling can be found in [4].

After the fading channel, as shown in Fig. 6, white Gaussian noise (WGN) is added to simulate the effect of overall interference in the system, including thermal noise, intra-cell (also referred as inner-cell) and inter-cell (outer-cell) interferences. Note that the dominating interference in CDMA systems tends to be inter-cell interference which is mainly due to the system having a unity reuse^9^ factor (if traffic is very heavy in adjoining cells). The carrier-to-interference ratio (*C/I*) is often used to denote a combination of intra-cell and inter-cell interference, and it can have values of 0 dB or less. In other words, the aggregate energy of the interference from neighboring cells may be higher than the energy of the desired signal.

## 5. Receiver

**Fig. 7:**
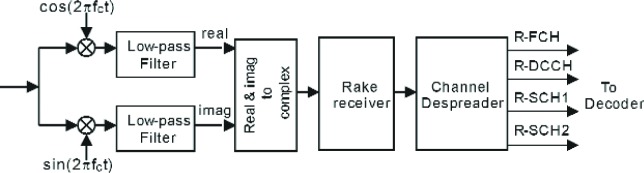
Receiver model for the cdma2000 system.

The receiver for the reverse link model consists of a rake receiver followed by a channel despreader. Figure 7 illustrates the block diagram of the receiver. The rake receiver attempts to collect the dispersed signal energy resulting from the multiple propagation paths between the transmitter and receiver. The rake receiver can therefore significantly reduce the effect of multipath fading.

As shown in Fig. 7, after demodulation (down-conversion to the base-band frequency from the carrier frequency), the low-pass filter, which is the same as the transmit filter, is applied to remove unnecessary noise and interference from the received signal. The output of the filter is then fed into the rake receiver. The rake receiver consists of several rake fingers and a combiner. Since the received signal is a composite signal made up of delayed versions of the transmitted signal with different attenuations, each rake finger is intended to focus on one of the multiple paths in the demodulation process. In this way, the rake receiver can create the output with a higher signal-to-noise ratio by combining the outputs of the rake fingers as compared with a single finger, i.e,. non-rake receiver. It is important for the rake receiver to estimate the channel coefficients (e.g., attenuation and phase) of each path to appropriately combine the outputs of the rake fingers (see Sec. 5.1). For the purpose of comparison, we also developed an ideal rake receiver, which assumes that the perfect (precise) channel information is available at the receiver. Since the ideal receiver can provide the best achievable performance, it can be used to evaluate and measure the relative performance of any practical (non-ideal) rake receiver. Details of designing the rake receiver will be discussed in succeeding sections.

### 5.1 Rake Receiver Design

The receiver may detect and combine up to *N* replicas of transmitted signals by using a rake receiver with *N* rake fingers. The number of available paths (replicas of the transmitted signals) at the receiver depends on the bandwidth of the transmitted signal as well as the characteristics of the propagation channel because the signal with a larger bandwidth has higher time resolutions. Therefore, spreading rate 3 can generally have a larger number of resolvable paths at the receiver than spreading rate 1. We designed a rake receiver with four fingers for spreading rate 1 (1.2288 Mcps), while a rake receiver with six fingers was utilized for spreading rate 3 (3.6864 Mcps).

**Fig. 8:**
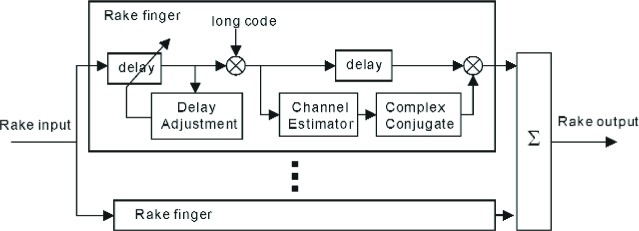
Rake receiver.

Figure 8 illustrates the rake finger structure. Each rake finger exploits the unmodulated pilot signal on the R-PICH to estimate the channel coefficients. Since the pilot signal is known at the receiver, the channel coefficients can be estimated by simply removing the spreading sequences (quadrature spreading sequence and Walsh sequence for the R-PICH). The pilot signal is also used to adjust the timing to track the aimed path (see Sec. 5.3). The rake receiver uses the complex conjugate values of the channel coefficients when combining the outputs from the fingers. A method known as maximum ratio combining is used to maximized the signal-to-noise ratio, provided that the noise (including interference) at each finger is an independently and identically distributed Gaussian random variable.

### 5.2 Channel Estimation

Since R-PICH carries the unmodulated signal spread by the long code, the output of the long code despreader can be used to estimate the channel coefficients at each rake finger. It is important to estimate the channel as accurately as possible to increase the signal-to-noise ratio effectively when combining the outputs of rake fingers. There are two popular approaches to enhance the accuracy of estimation. One is to increase the signal-to-noise ratio by using a longer observation duration. The other is to apply interference cancellation to suppress multipath as well as inter-cell or intra-cell interferences. Currently, there are a number of interference cancellation techniques that can be utilized for CDMA systems. Unfortunately, most adaptive or non-adaptive linear interference cancellation techniques, such as MMSE (Minimum Mean Squared Error) [8] or CMA (Constant Modulus Algorithm) [8] are not suitable for the cdma2000 system. This is because the interference in cdma2000 cannot be considered as a cyclostationary process (i.e., on a symbol-by-symbol basis) as the cdma2000 system deploys a very long spreading code. However, methods such as ZF (zero forcing) equalizer [8] may found to be more suitable if all the delays of the paths can be accurately estimated. Nevertheless, such a method would suffer from high computation cost (e.g., matrix inversion) in estimating the filter coefficients within a symbol period.

**Fig. 9:**
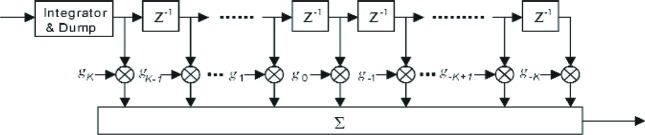
Channel estimator.

In our simulation model as shown in Fig. 9, we have used a simple linear filter without deploying any interference cancellation techniques for channel estimation. To cancel the effect of the other channels (R-FCH, R-DCCH, R-SCHs), the estimator first integrates the output of the long code despreader over sixteen chips on the R-PICH, which are here referred to as a pilot symbol. This is equivalent to taking a correlation with the “all one” Walsh functions. This estimator uses a simple filter with (2*K*+1) taps (i.e., *K* preceding pilot symbols, *K* succeeding pilot symbols, and current pilot symbol) to estimate the channel coefficients for the current symbol. In designing the filter, the length of observation (2*K*+1) should be as large as possible if the channel is approximately invariable (low mobility). At the same time, at higher velocities (high mobility) where the channel changes dramatically, a shorter length filter should be deployed. In addition, depending on the channel variation, the filter taps (*g*_0_, *g*_1_, …, *g_K_*) should also be selected in accordance with channel conditions. For instance, when the channel variation is slow (within 2*K*+1 consecutive symbols), a filter with equal weights can improve the estimation accuracy. The performance evaluation of such filter under various testing conditions will be discussed in Sec. 7.

### 5.3 Delay Adjustment

**Fig. 10:**
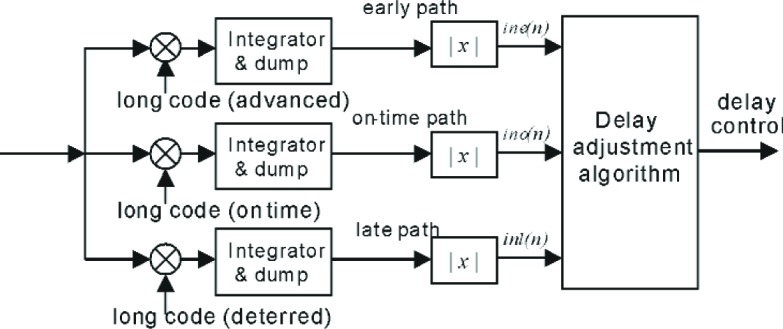
Delay adjustment block.

Since the propagation channel is time-varying in general, the rake finger need to keeps track of its change adaptively. Figure 10 illustrates the delay adjustment block. The pilot symbols are used to adjust the delay at each finger. As shown in this figure, within each finger there are three paths, an early path, an on-time path and a late path, which are correlated with the same long code with slightly different phases (starting points). In each of these paths the pilot Walsh sequence is removed and then integrated over the symbol duration. The magnitudes of the outputs are used to calculate a timing correction once per frame.

At the end of the frame, the delay adjustment block evaluates the following value,

$$\lambda =\frac{Y_{1}(n+1)-Y_{e}(n+1)}{Y_{0}(n+1)+1}$$

where *Y*_e_(*n*+1), *Y*_0_(*n*+1), and *Y*_l_(*n*+1) are calculated recursively at each symbol time *n*, and are given by

$$\begin{array}{l} Y_{e}(n+1)=(1-A)Y_{e}(n)+A\;in_{e}(n)\\ Y_{0}(n+1)=(1-A)Y_{0}(n)+A\;in_{0}(n)\\ Y_{1}(n+1)=(1-A)Y_{1}(n)+A\;in_{1}(n) \end{array}$$

where *A* is a predefined value between 0 and 1. Depending on the value of *λ*, the receiver may adjust the delay (see Fig. 8) according to the following rule.

$$\begin{array}{l} if|\lambda |>threshold,\text{then}\\ \;\text{if}\;\lambda <0\;t=+1\\ \;\text{else}\;t=-1\\ \text{else}\;t=0 \end{array}$$

where *t* is the timing correction value and *threshold* is a predefined value, which decides whether a timing correction is required.

### 5.4 Diversity Reception

**Fig. 11:**
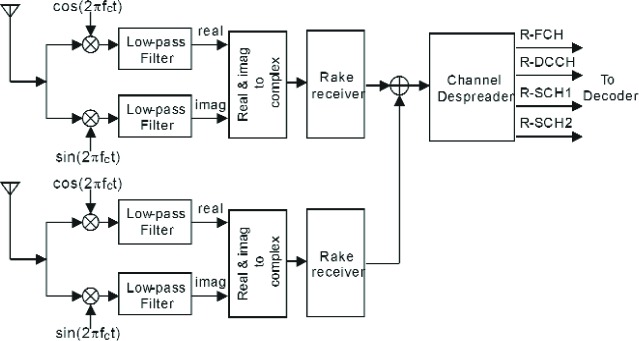
Receiver with dual antenna diversity.

In addition to a rake receiver, it is also useful to apply diversity reception with multiple receiver antennas at a base station to improve the system capacity. We have also considered a straightforward receiver model with dual diversity reception according to Fig. 11, which has two antennas and rake receivers. In this model, the outputs from two rake receivers are combined and then fed into the channel despreader. The operations at each block are exactly the same as one without diversity reception as described in the previous sections. Since the combined signal from two rake receivers has a higher signal-to-noise ratio, the diversity receiver can improve the performance significantly under the fading environments.

In this model, the outputs from the totally 2*N* rake fingers are combined together. It may be preferable to use a single rake receiver instead of two rake receivers in terms of implementation cost. Since this receiver is almost equivalent to using a rake receiver with 2*N* rake fingers, it is advantageous to use a single rake receiver with antenna diversity. For instance, *N*/2 rake fingers in the rake receiver are assigned to the low-pass filter out second antenna.

As a result, the rake receiver picks up totally *N* paths from two receiver antennas instead of 2*N* paths.

### 5.5 Channel Despreading

**Fig. 12:**
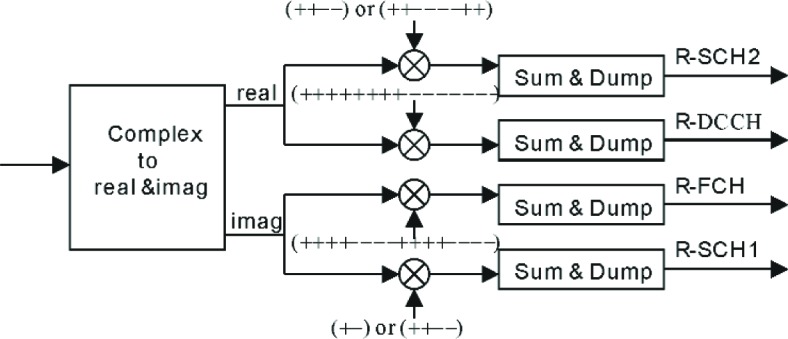
Channel despreader.

Following the rake receiver is the channel despreader. Its block diagram is shown in Fig. 12. Thanks to the orthogonal properties of Walsh functions, the channel despreader can separate the output signal from the rake receiver into individual channels using Walsh functions. The complex output signal from the rake receiver is first separated into real and imaginary parts. Note that the real part contains the R-SCH2 and DCCH, and the imaginary part contains the R-FCH and R-SCH1 (see Fig. 5). Then the (coded) data stream on each channel can be recovered by taking the correlation between the real (or imaginary) part signal and the same Walsh function used at the transmitter, as shown in Fig. 12. Outputs from the despreader are then fed into the channel decoder which will be discussed in the following section.

### 5.6 Base Station Decoding

**Fig. 13:**
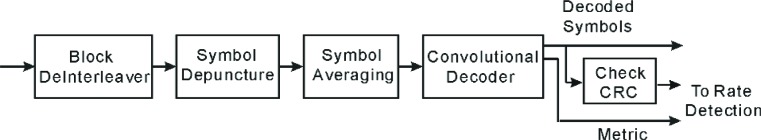
Decoder structure on the Reverse Traffic Channel.

The reverse link decoder consists of an R-FCH decoder, R-SCH decoder, and R-DCCH decoder. The R-SCH decoder is actually made up of the R-SCH1 and R-SCH2 decoders. The operations of these decoders are basically common and are the reverse operations of the encoders. Figure 13 shows the general structure of the decoder. After storing the outputs from the channel despreader for the frame duration, the deinterleaver rearranges the order in the opposite way to the interleaver. Then zeros are inserted into the data stream according to the puncturing pattern at which the encoder was supposed to puncture. Then to compensate symbol repetition, the data stream may be averaged over the length of the repetition factor. After that, data can be recovered by the maximam likelihood decoder, a.k.a. Viterbi decoder. The decoded symbols are then verified by using CRC bits. Note that the R-FCH decoder requires an additional operation (i.e., blind rate detection) to estimate the data rate because such information is not explicitly transmitted (note that R-FCH can use transmission at variable rates). Bear in mind that rate detections are not necessary for the R-SCH because the rates are fixed.

The data rate on the R-FCH is variable, with the ability to change on a frame-by-frame basis. Since the rate information is not explicitly transmitted, the decoder must estimate the data rate based on the received signal. Note that no particular rate decision algorithm has been described in the cdma2000 specification. We have developed an R-FCH decoder consisting of four convolutional decoders. Each decoder is designed to decode one of four possible data rates. At this stage, there are four candidates for the R-FCH data stream. The receiver chooses one based on the CRCs and the Viterbi metrics. The Viterbi metric indicates the likelihood that the decoded data is correct. In the event of a single CRC success, the rate that triggers the success is chosen. It is necessary to have another decision measure, both as a backup and to handle the lower rates of Radio Configuration 1, which does not include CRCs. For cases of zero or multiple successes, the data with highest Viterbi metric is selected.

## 6. Link Budget

The reverse link budget calculation is based on the equations specified in the cdma2000 Physical Layer Proposal [1] for allocating power to the CDMA channels. In the equations, mean power is referenced to the nominal CDMA Channel bandwidth of 1.23 MHz for spreading rate 1 (*N* = 1) and 3.69 MHz for spreading rate 3 (*N* = 3). For simplicity, the term “Code Channel” is used here to represent the R-FCH, R-SCH1, R-SCH2, or R-DCCH. The output (transmit) power of each Code Channel is set at the mobile station relative to that of the R-PICH. Therefore, the first task is to determine the output power of the R-PICH, *P*_pilot_, using open loop power estimation [1]. Then, the output power of every Code Channel, *P*_code_, can be calculated, based on the *P*_pilot_ and the stored parameters in the mobile station.

### 6.1 Pilot Output Power Calculation

The equation for calculating the mean pilot channel output power when transmitting on the Reverse Traffic Channel with Radio Configurations 3–6, is given by

$$\begin{array}{l} P_{\text{pilot}}=-\text{Mean}\;\text{input}\;\text{power}\\ \;+\;\text{Offset}\;\text{power}\\ \;+\;NOM\text{\_}PWRS-(16\times NOM\text{\_}PWR\text{\_}EXTS)\\ \;+\;INIT\text{\_}PWRS\\ \;+\;RL\text{\_}GAIN\text{\_}ADJS \end{array}$$ (1)

where *P*_pilot_ is the mean pilot channel output power and is expressed in dB: relative to 1 mW (i.e., 10 log_10_ *P*_pilot_/1 mW). Mean input power is the received power at the mobile station’s antenna connector from the base station. Offset power is a fixed value of −84.5 dB or −79.5 dB for spreading rate 1 or 3, respectively. *NOM_PWRS* is the nominal transmit power adjustment. *NOM_PWR_EXTS* is the extended nominal transmit power offset. These two parameters are correction factors to be used by the mobile station in the open loop power estimate, initially applied on the Access Channel [1]. *INIT_PWRS* is the adjustment that is made to the first Access Channel probe (i.e., first attempt) so that it can be received somewhat below the level required for it to be normally detected by the base station. This conservatism partially compensates for occasional, partially decorrelated path losses between the Forward CDMA Channel and the Reverse CDMA channel.

The purpose of having both an *INIT_PWRS* and a *NOM_PWR_EXTS* is to distinguish between their uses. If *INIT_PWRS* were 0, then *NOM_PWRS* - (16×*NOM_PWR_EXTS*) would be the correction that should provide the correct received power at the base station. The *NOM_PWRS* - (16×*NOM_PWR_EXTS*) correction allows the open loop estimation process to be adjusted for different operating environments. The total range of the *NOM_PWRS* - (16×*NOM_PWR_EXTS*) correction is from −24 dB to 7 dB. The range of the *INIT_PWRS* is from −16 dB to 15 dB, with a nominal value of 0 dB.

*RL_GAIN_ADJS* is the gain adjustment of the channel relative to the last channel used (i.e., the Access Channel, the Enhanced Access Channel or the Reverse Common Control Channel) before the Reverse Traffic Channel is operating. As a whole, it can be seen that *P*_pilot_ will be adjusted to a higher value as soon as the Mean input power drops or vice versa, provided the stored parameters mentioned above are kept constant.

### 6.2 Code Channel Output Power Calculation

Based on the output power of the R-PICH, *P*_pilot_ calculated in Eq. (1), the mobile station sets the output power of each Code Channel (R-FCH, R-SCH1, R- SCH2 or R-DCCH), *P*_code_, as follows:

$$\begin{array}{l} P_{\text{code}}=P_{\text{pilot}}\\ \;+\;0.125\times \;(Nominal\text{\_}Attribute\text{\_}Gain-Multiple\text{\_}Channel\text{\_}Adjustment\text{\_}Gain)\\ \;+\;RLAGIN\text{\_}TRAFFIC\text{\_}PILOTS\\ \;+\;RLGAIN\text{\_}SCH\text{\_}PILOT[i]S \end{array}$$ (2)

where *Nominal_Attribute_Gain* represents the nominal Code Channel power relative to the *P*_pilot_. This parameter is listed in Table 4 for each data rate in rateset 1 and rateset 2 supported by the mobile station.^10^ Note that the values of *Nominal_Attribute_Gain* and Pilot_Reference_Level are integers, specified in units of 0.125 dB. For example, if the R-SCH1 is operating at 19.2 kbit/s (i.e., data rate 4, rateset-1) the *Nominal_Attribute_Gain* would be 50, which equals 6.25 dB. It can be seen in Table 4 that the *Nominal_Attribute_Gain* increases with the data rate in kbit/s.

*Pilot_Reference_Level* is similarly listed in Table 6.1. *Pilot_Reference_Level* is used to determine the *Multiple_Channel_Adjustment_Gain.* If the mobile station is transmitting on only one Code Channel in addition to the R-PICH, then the *Multiple_Channel_Adjustment_Gain* shall be set to zero. If the mobile station is transmitting on two or more Code Channels in addition to the R-PICH, then the *Multiple_Channel_Adjustment_Gain* is calculated as follows:

1. Select the Code Channel having the highest *Pilot_Reference_Level* as Max Channel
2. On the Max_Channel, *Multiple_Channel_Adjustment_Gain* is 0.
3. On all other Code Channels, *Multiple_Channel_Adjustment_Gain* = *Pilot_Reference_Level* of the Max Channel-*Pilot_Reference_Level* of that particular Code Channel.

For instance, when the mobile station is transmitting with Radio Configuration 5 (rateset 1) on the R-FCH at data rate 0, R-DCCH at data rate 0, R-SCH1 at data rate 4, and R-SCH2 at data rate 5, *Multiple_Channel_Adjustment_Gain* for every Code Channel can be derived from its associated *Pilot_Reference_Level* as shown in Table 5. In this case, R-SCH2 is the Max Channel.

The output power for the Code Channel is further adjusted by *RLGAIN_TRAFFIC_PILOTS*, which is the gain adjustment of the Reverse Traffic Channel relative to the R-PICH. Similarly, *RLGAIN_SCH_PILOT[i]S* is the gain adjustment of the R-SCHi relative to the R-PICH, where *i* is 1 or 2. Both *RLGAIN_TRAFFIC_PILOTS* and *RLGAIN_SCH_PILOTS* are the mobile stations stored parameters. Note that *RLGAIN_SCH_PILOT[i]S* is valid only for the R-SCHs.

### 6.3 Signal-to-Noise Ratio Calculation

So far, we have described how to calculate the average output powers for the Reverse Traffic Channel. The next step is to calculate the signal-to-noise ratio, *E*_b_ / *N*_t_, which is defined as the ratio of the combined received energy per bit to the effective noise power spectral density on the R-PICH, R-FCH, R-SCH1, R-SCH2, or R-DCCH at the receiver base station antenna connector [9].

The *E*_b_ / *N*_t_ on the Traffic Channel is calculated by summing the signal-to-noise ratios over the individual paths. For example, for the R-FCH, the *E*_b_ / *N*_t_ is given by

$${\left(E_{b}/N_{t}\right)}_{\text{R-ECH}}=\sum_{i=1}^{N}\frac{\left[E_{b}{\left(i\right)}_{\text{R-ECH}}\right]}{N_{t}(i)}$$ (3)

where *N* is the number of reflected paths. The calculation of *E*_b_ (*i*) and *N*_t_ (*i*) are given as follows,

$${\left[E_{b}(i)\right]}_{\text{R-FCH}}=\frac{path\text{\_}loss\cdot pwr\text{\_}fch\cdot pwr(i)}{bit\text{\_}rate}$$ (4)

$$N_{t}(i)=I_{0}\sum_{j\neq i}pwr(j)+N_{t}$$ (5)

where *path_loss* is the average pass loss for the first path and *pwr* (*i*) is the average power for the *i*th path relative to the first path. For instance, as shown in Fig. 6, for the six paths IMT - 2000 vehicular Model-A these powers are defined as: *pwr* (1) = 0 dB, *pwr* (2) = −1 dB, *pwr* (3) = −9 dB, *pwr* (4) = −10 dB, *pwr* (5) = −15 dB, and *pwr* (6) = −20 dB. The *pwr_fch* is the output power on the R-FCH calculated from Eq. (1). The *N*_t_ is the noise plus inter-cell and intra-cell interference power in W/Hz, which is applied to obtain the variance of the additive white Gaussian noise (AWGN) in the SPW model.

The *I*_0_ is defined as the total power spectral density of a single user in the cell and is calculated from

$$I_{0}=\frac{total\text{\_}power\cdot path\text{\_}loss}{chip\text{\_}rate}$$ (6)

where *total_power* is total output (transmit) power in W and *chip_rate* is chip rate in Hz.

## 7. Model and Evaluation

Our main objective in this section is to evaluate the effect of the receiver design and diversity on the performance of cdma2000 reverse-link physical layer. A detailed description of the rake receiver design, including an ideal rake receiver, has been given in Sec. 5. The IMT-2000 Vehicular Model-A, shown in Fig. 5, was used as the propagation channel model together with AWGN to simulate thermal noise plus inter-cell and intra-cell interference. In the SPW simulation model, each chip is over-sampled by a factor of 8. As a low-pass filter at the transmitter and receiver, the square-root raised-cosine (SRRC) filter with roll-off factor of 0.22 was employed. In our model, it is assumed that each finger in the rake receiver has perfect synchronization with the corresponding path. The carrier frequency was set at 1.9 GHz. For each measurement, the mean output power on each channel was calculated from the reverse link model in accordance with the link budget (see Sec. 6). All measurements carried out in this paper are based on a single user transmission using Radio Configurations 5 and 6. In addition, the initial values and relative gain adjustments in the link budget were set to zero (see Sec. 6). As discussed earlier, the mean pilot channel power was first calculated by the SPW model’s link budget by specifying the Received Power Spectral Density (PSD) at the mobile station’s antenna connector.

**Fig. 14:**
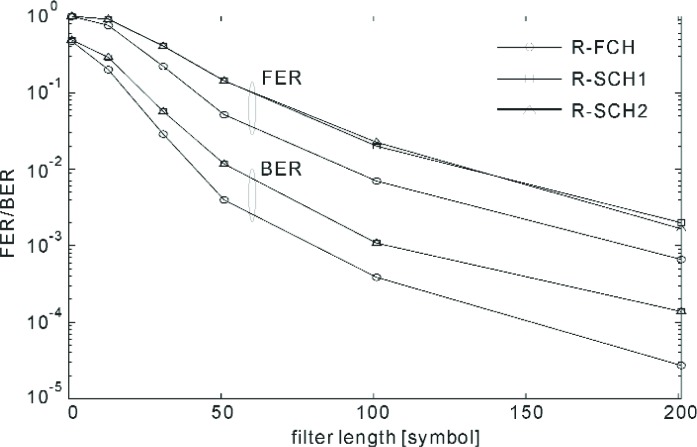
FER/BER versus filter length (velocity = 100 km/h).

**Fig. 15:**
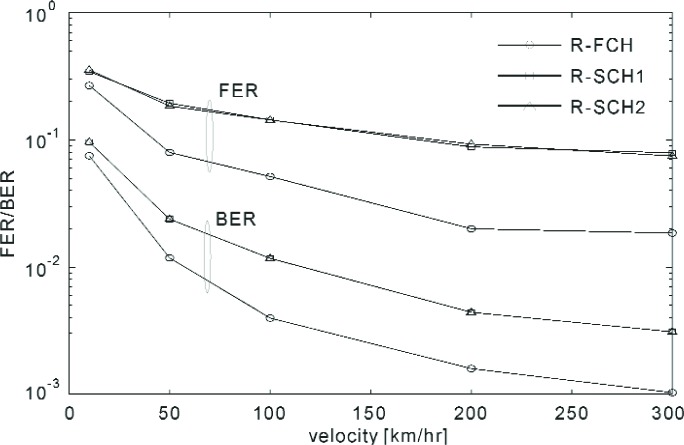
FER/BER versus velocity (filter length = 51).

These measurements were performed according to the test parameters that are tabulated in Tables 6–9. The first task was to assess the effect of the filter length (see Fig. 11) on the performance of the channel estimator. Figures 14 and 15 show the receiver performance versus the filter length. In these experiments, we used the test parameters shown in Table 6 for Radio Configuration 5. The noise plus interference power (see Sec. 6.3) is fixed at −78 dB (relative to 1 mW). Figure 14 shows the FER/BER performance with different filter lengths, where the mobile station’s velocity is 100 km/h. This figure indicates that as the filter length becomes longer, the FER/BER performance improves considerably. Figure 15 shows the FER/BER performance with different mobile station’s speeds, where the filter length is set to 51 symbols with uniform weights. As shown in this figure, the performance depends largely on the velocity of the mobile station. In fact, the FER/BER performance improves as the velocity increases, despite the fact that the performance of the channel estimator is expected to suffer at higher velocities. Such behaviour is mainly to do with the contribution of the forward error correction code and the interleaver, which are more effective under faster channel variations; whereas at a slower fading rate the channel does not change noticeably within 51 symbols.

**Fig. 16:**
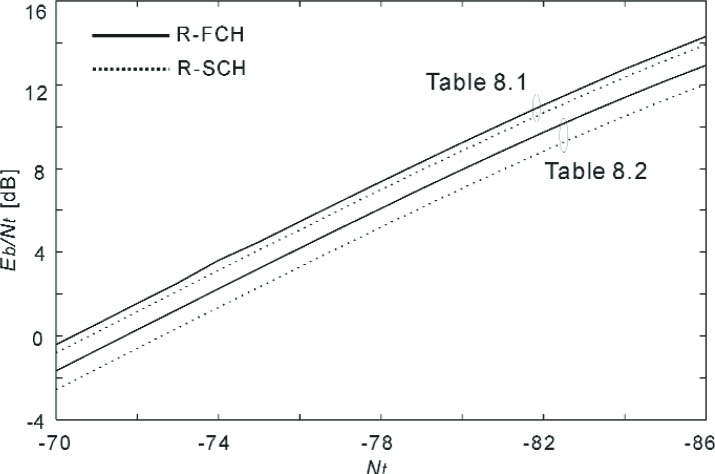
Calculated *E*_b_ / *N*_t_ versus *N*_t_ for Radio Configuration 5.

**Fig. 17:**
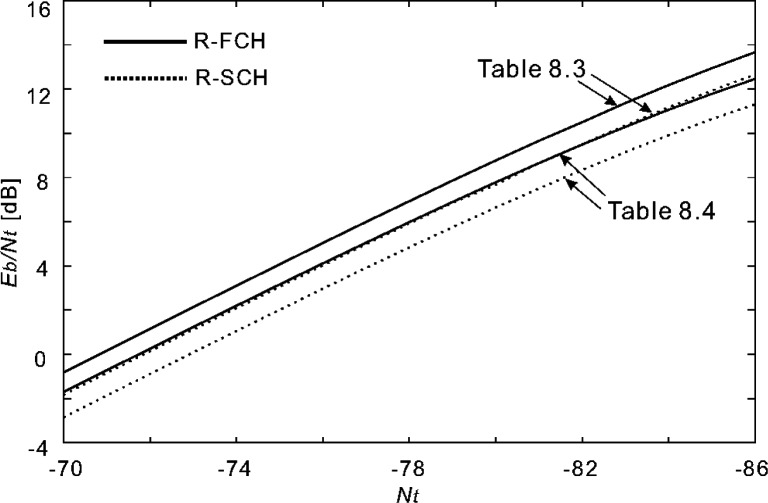
Calculated *E*_b_ / *N*_t_ versus *N*_t_ for Radio Configuration 6.

Having evaluated the effect of the channel estimator’s filter length, in the following experiments we used 101 pilot symbols (*K* = 50 with uniform weights) for channel estimation. Figures 16–17 show the calculated *E*_b_ / *N*_t_, versus the noise spectral density *N*_t_ with these test parameters for the Radio Configurations 5 and 6, respectively.

**Fig. 18:**
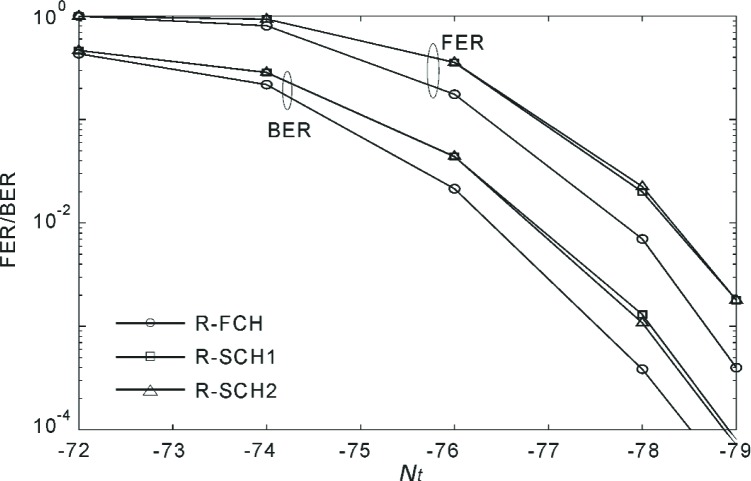
FER and BER versus *N*_t_ for Radio Configuration 5 (Table 6).

Figure 18 shows the FER and BER versus *N*_t_ for Radio Configuration 5 according to the test parameters shown in Table 6. We observe that the R-FCH performs better than the R-SCHs despite the fact that the R-FCH has smaller transmit power (*P*_code_), as indicated in Table 6. One possible explanation is that the R-FCH suffers from smaller interference because of a larger spreading gain (note that spreading gain is defined as a ratio of chip rate over bit rate). This can be verified by the fact that the R-FCH has higher signal-to-noise ratio than the R-SCH for the same *N*_t_ (see Fig. 16). Another possible explanation is that the R-FCH is more robust against fading because the same symbol appears twice (i.e., repetition factor 2) in the interleaver on the R-FCH, while the repetition factor is one on the R-SCH1 in this case. Since the R-SCH1 and R-SCH2 have the same bit rates and same *E*_b_ / *N*_t_, they have very similar performance.

**Fig. 19:**
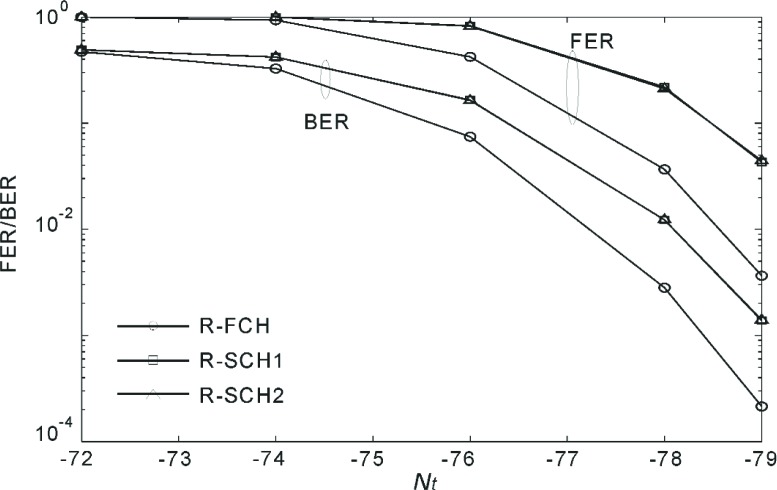
FER and BER versus *N*_t_ for Radio Configuration 5 (Table 7).

Figure 19 similarly shows the FER and BER for Radio Configuration 5 with the parameters in Table 7. Since the *E*_b_ / *N*_t_ is lower than that with the parameters in Table 6 for the same *N*_t_, the FER/BER performance also becomes slightly worse than those in Fig. 18. This is mainly because of the higher bit rates on the R-SCHs.

**Fig. 20:**
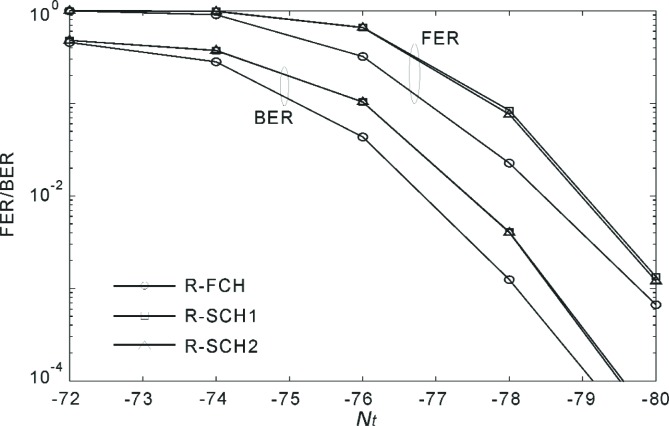
FER and BER versus for Radio Configuration 6 (Table 8).

**Fig. 21:**
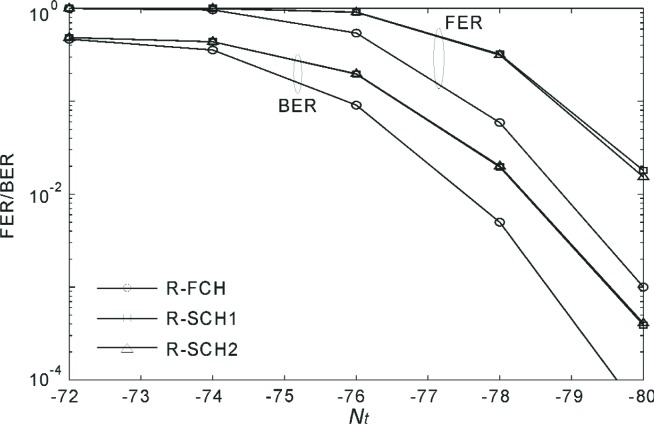
FER and BER versus for Radio Configuration 6 (Table 9).

Figures 20–21 show the FER and BER performances for Radio Configuration 6 with the test parameters defined in Tables 8–9, respectively. We notice the same tendencies observed in Figs. 18–19. Due to higher bit rates (smaller spreading gains), the *E*_b_ / *N*_t_ on the R-SCHs is smaller by about 1 than the R-FCH as shown in Fig. 17. Consequently, the R-FCH provides better error rate performances than the R-SCHs as we observed in the case of Radio Configuration 5.

**Fig. 22:**
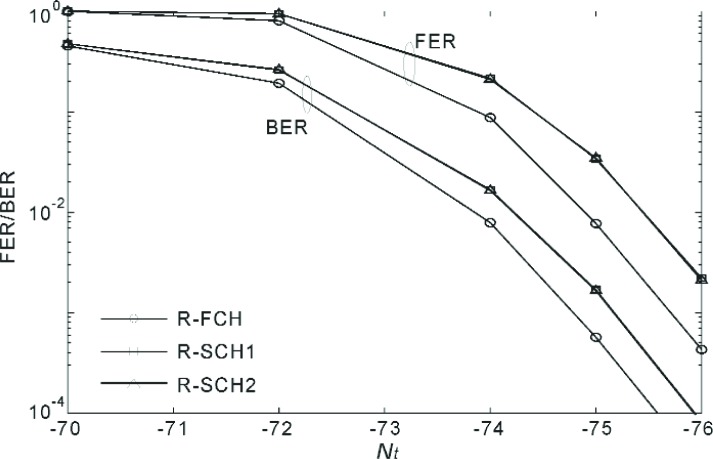
FER and BER versus *N*_t_ with diversity for Radio Configuration 5 (Table 6).

Figure 22 shows the FER and BER with diversity reception discussed in Sec. 5.4, using Radio Configuration 5. Comparing the results with Fig. 18 (non-diversity receiver), as expected we observe that the diversity receiver performs significantly better than the non-diversity receiver. As can be seen in this figure, the difference between these receivers becomes more obvious as the signal-to-noise ratio increases.

**Fig. 23:**
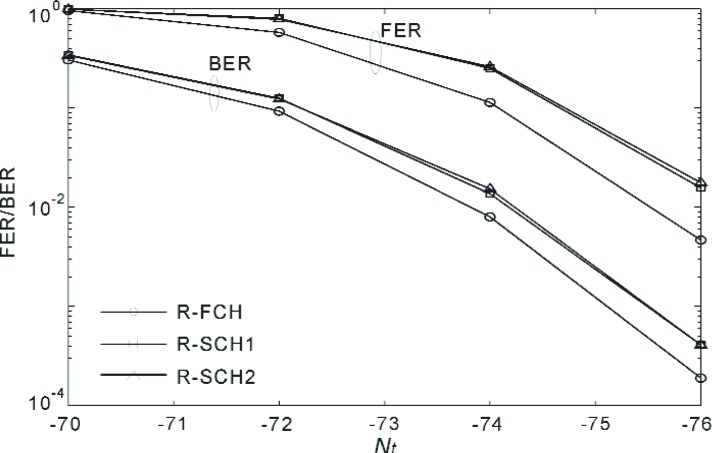
FER and BER versus *N*_t_ with ideal rake receiver for Radio Configuration 5 (Table 6).

Figure 23 shows the performance with an ideal rake receiver for Radio Configuration 5 with the test parameters defined in Table 6. As stated in Sec. 5, the ideal receiver uses the actual channel gains when combining the rake fingers’ outputs instead of the channel estimator. The results of the ideal receiver suggest an achievable best performance by the rake receiver with a channel estimator. Comparing the results in Fig. 23 and Fig. 18, we notice that the difference in terms of *E*_b_ / *N*_t_ (or Nt) is about 2.5 dB between the ideal and non-ideal receivers. This performance degradation is mainly caused by interference from the other channels and other paths as well as the noise. As shown in Sec. 5.2, it is possible to improve the performance by using the longer filter length for channel estimator.

## 8. Conclusion

Having previously developed the cdma2000 forward link and the multicarrier models, the next challenge was to develop the cdma2000 reverse link. CDMA systems rely heavily on strict power control to effectively manage the channel capacity. Each mobile unit has its own power control to handle the path loss and aggregate interference. In the cdma2000 reverse link a tight budget link has been specified to handle the power allocation on every transmitted channel. Thus, one of our most important objectives in developing the reverse link has been the implementation and inclusion of the link budget in our model. The link budget has been a crucial factor in performing our measurements according to the test environment and the link parameters specified by the standard.

In addition, as the standard practice is not to recommend any specific design for the receiver, our next challenge was to develop a suitable receiver for our model. Bear in mind that the efficiency of a receiver can have a significant effect on the overall performance of a CDMA system. We have therefore implemented the rake receiver with a channel estimator and optional antenna diversity. For the purpose of comparison, we have also implemented an ideal rake receiver. In our experiments, the performance of these receivers has been evaluated and compared.

Finally, the viability of the cdma2000 reverse link has undergone extensive testing to revalidate the model. However, considering the complexity, the sheer size of the simulation model, and the limited manpower, there is no way that we can guarantee the full accuracy of the model. Nevertheless, we plan to continue our efforts to further test and revalidate the model and perform further research and investigation to extend the model in conjunction with recent TIA/EIA IS2000 revisions.

## Figures and Tables

**Fig. 24:**
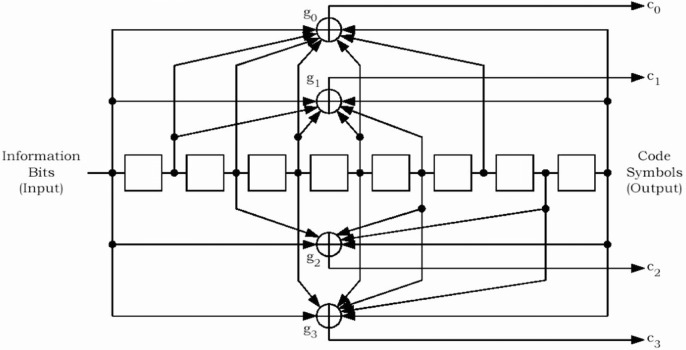
Rate 1/4 convolutional encoder.

**Fig. 25:**
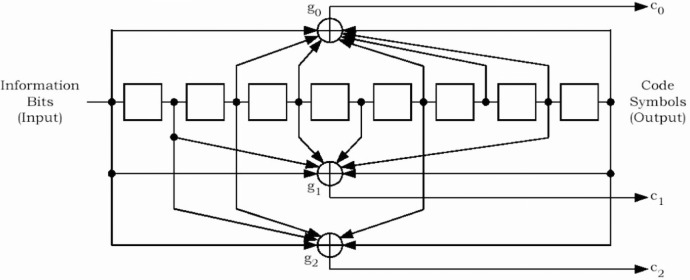
Rate 1/3 convolutional encoder.

**Fig. 26:**
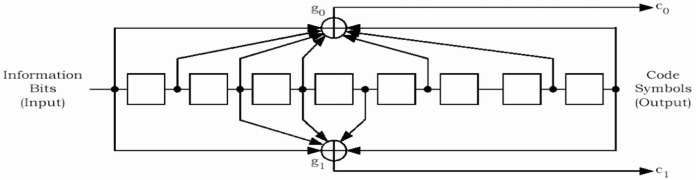
Rate 1/2 convolutional encoder.

**Table 1:** Radio Configuration characteristics for the R-FCH, R-SCH and R-DCCH

Radio configuration	Associated spreading rate	Rateset	Data rates (kbit/s)		
			R-FCH	R-SCH	R-DCCH
3	1	1	1.5, 2.7, 4.8, 9.6	1.5, 2.7, 4.8, 9.6, 19.2, 38.4, 76.8, 153.6, 307.2	9.6
4	1	2	1.8, 3.6, 7.2, 14.4	1.8, 3.6, 7.2, 14.4 28.8, 57.6, 115.2, 230.4	14.4
5	3	1	1.5, 2.7, 4.8, 9.6	1.5, 2.7, 4.8, 9.6, 19.2, 38.4, 76.8, 153.6 307.2, 614.4	9.6
6	3	2	1.8, 3.6, 7.2, 14.4	1.8, 3.6, 7.2, 14.4, 28.8, 57.6, 115.2, 230.4, 460.8,1036.8	14.4

**Table 2:** Framing and encoding parameters on the R-FCH and R-SCH for Radio Configuration 5

Radio config.	Data rate (kbit/s)	Number of bits per frame					Repetition factor	Basic code rate	Puncturing rate	Interleaver size
		Total	Reserved	Info.	CRC	Tail				
	614.4	12288	0	12264	16	8	1	1/3	None	36864
	307.2	6144	0	6120	16	8	1	1/3	None	18432
	153.6	3072	0	3048	16	8	1	1/4	None	12288
	76.8	1536	0	1512	16	8	1	1/4	None	6144
5	38.4	768	0	744	16	8	1	1/4	None	3072
	19.2	384	0	360	16	8	1	1/4	None	1536
	9.6	192	0	172	12	8	2	1/4	None	1536
	4.8	96	0	80	8	8	4	1/4	None	1536
	2.7	54	0	40	6	8	8	1/4	1 of 9	1536
	1.5	30	0	16	6	8	16	1/4	1 of 5	1536

**Table 3:** Walsh functions for reverse CDMA channels

Channel type	Walsh function
Reverse pilot channel	***W***_0_^32^
Reverse dedicated control channel	***W***_8_^16^
Reverse fundamental channel	***W***_4_^16^
Reverse supplemental channel 1	***W***_1_^2^ or ***W***_2_^4^
Reverse supplemental channel 2	***W***_2_^4^ or ***W***_6_^8^

**Table 4:** Nominal_Attribute_Gain and Pilot_Reference_Level

Input data rate	Data rate (in kbit/s)		*Nominal_Attribute_Gain*		*Pilot_Reference_Level*	
	rateset 1	rateset 2	rateset 1	rateset 2	rateset 1	rateset 2
0	9.6	14.4	30	44	0	3
1	4.8	7.2	−2	15	0	3
2	2.7	3.6	−22	−13	0	3
3	1.5	1.8	−47	−42	0	3
4	19.2	28.8	50	56	1	7
5	38.4	57.6	60	72	11	14
6	76.8	115.2	72	80	21	28
7	153.6	230.4	84	88	36	43
8	307.2	460.8	96	104	54	61
9	614.4	1036.8	112	128	68	83

**Table 5:** Example of Multiple_Channel_Adjustment_Gain calculation

Code channel	Input data rate	*Pilot_Reference_Level*	*Multiple_Channel_Adjustment_Gain*
R-FCH	0	0	11−0 = 11
R-DCCH	0	0	11−0 = 11
R-SCH1	4	1	11−1 = 10
R-SCH2	5	11	0

**Table 6:** Test Parameters and mean power values for Radio Configuration 5

Channel	Information rate kbit/s	Path loss dB	Vehicular Speed km/h	*P*_code_ dB (relative to 1 mW)	Received PSD dB (relative to 1 mW)
R-PICH	Unmodulated	100	100	−3.0	−76.5
R-FCH	9.6	100	100	0.625	−76.5
R-SCH1	19.2	100	100	3.25	−76.5
R-SCH2	19.2	100	100	3.25	−76.5
R-DCCH	9.6	100	100	0.625	−76.5

**Table 7:** Test Parameters and mean power values for Radio Configuration 5

Channel	Information rate kbit/s	Path loss dB	Vehicular Speed km/h	*P*_code_ dB (relative to 1 mW)	Received PSD dB (relative to 1 mW)
R-PICH	Unmodulated	100	100	−3.0	−76.5
R-FCH	9.6	100	100	−0.625	−76.5
R-SCH1	38.4	100	100	4.5	−76.5
R-SCH2	38.4	100	100	4.5	−76.5
R-DCCH	9.6	100	100	−0.625	−76.5

**Table 8:** Test Parameters and mean power values for Radio Configuration 6

Channel	Information rate kbit/s	Path loss dB	Vehicular Speed km/h	*P*_code_ dB (relative to 1 mW)	Received PSD dB (relative to 1 mW)
R-PICH	Unmodulated	100	100	−3.0	−76.5
R-FCH	14.4	100	100	2.0	−76.5
R-SCH1	28.8	100	100	4.0	−76.5
R-SCH2	28.8	100	100	4.0	−76.5
R-DCCH	14.4	100	100	2.0	−76.5

**Table 9:** Test Parameters and mean power values for Radio Configuration 6

Channel	Information rate kbit/s	Path loss dB	Vehicular Speed km/h	*P*_code_ dB (relative to 1 mW)	Received PSD dB (relative to 1 mW)
R-PICH	Unmodulated	100	100	−3.0	−76.5
R-FCH	14.4	100	100	1.15	−76.5
R-SCH1	57.6	100	100	6.0	−76.5
R-SCH2	57.6	100	100	6.0	−76.5
R-DCCH	14.4	100	100	1.15	−76.5

**Table 10:** Generator functions for different encoding rates

Encoding rate	g0 (octal)	g1 (octal)	g2 (octal)	g3 (octal)
1/4	765	671	513	473
1/3	557	663	711	N/A
1/2	753	561	N/A	N/A

## Footnotes

1 IS-95 is EIA (Electric Industry Association) Interim Standard 95. This is a digital mobile telephony standard based on CDMA technology.

2 Chip is defined as a unit after spreading every data symbol.

3 Certain commercial equipment, instruments, or materials are identified in this paper to foster understanding. Such identification does not imply recommendation or endorsement by the National Institute of Standards and Technology, nor does it imply that the materials or equipment identified are necessarily the best available for the purpose.

4 Reverse link and forward link SPW models have been developed at NIST: http://w3.antd.nist.gov/wctg/3G/.

5 A method of organization for communication protocols in cdma2000 is made of three layers (layers 1,2, and 3). MAC sublayer is in layer 2 and implements the medium access protocol.

6 Maximal length linear feedback shift register sequence, a.k.a. m-sequence, is a type of pseudo random sequence generated by a linear feedback shift register. A m-sequence has the periodic auto-correlation function with two values.

7 IMT-2000 (International Mobile Telecommunication 2000) is a term used by the ITU to describe the third generation mobile telephony and standard.

8 Vehicular Model-A is a type of channel model used for simulating and evaluating an IMT-2000 system.

9 The reuse factor is the number of distinct communication channel sets used per cluster of cells. The unit means that every cell uses the same communication channel set.

## 9. Appendix A. Convolutional Coding

The generator function for each encoding rate (1/4, 1/3, or 1/2) is listed in Table 10. For rate 1/3, the generator function g3 is not applicable. For rate 1/2, g2 and g3 are not applicable. The code symbols are sent out so that the code symbol (c0) encoded with generator function g0 goes out first, the code symbol (c1) encoded with generator function g1 is second, and so forth if necessary.

The state of the convolutional encoder, upon initialization, is the all-zero state. The first code symbol that is outputed after initialization is a code symbol encoded with generator function g0. The encoders for the three different rates are illustrated in Figs. 24, 25, and 26. The constraint length is 9 for every encoder. Further details on convolutional coding can be found in [5]
